# Non-alcoholic Fatty Liver Disease Is Associated With Aortic Calcification: A Cohort Study With Propensity Score Matching

**DOI:** 10.3389/fendo.2022.880683

**Published:** 2022-05-16

**Authors:** Rong-Rong Zhu, Xu-Ping Gao, Min-Qi Liao, Yun-Feng Cui, Si-Xian Tan, Fang-Fang Zeng, Yan-Mei Lou, Chang-Yi Wang, Shan Xu, Xiao-Lin Peng, Shu-Hong Dai, Dan Zhao, Li Wang, Zhao Ping, Xiao-Yu Dai, Pin-Ning Feng, Li-Yuan Han

**Affiliations:** ^1^ Department of Pharmacy, Hua Mei Hospital, University of Chinese Academy of Sciences, Zhejiang, China; ^2^ Department of Public Health and Preventive Medicine, School of Medicine, Jinan University, Guangdong, China; ^3^ Department of Child & Adolescent Psychiatry, Peking University Sixth Hospital (Institute of Mental Health), National Clinical Research Center for Mental Disorders and NHC Key Laboratory of Mental Health (Peking University Sixth Hospital), Beijing, China; ^4^ Department of Health Management, Beijing Xiao Tang Shan Hospital, Beijing, China; ^5^ Department of Non-communicable Disease Prevention and Control, Shenzhen Nanshan Center for Chronic Disease Control, Shenzhen, China; ^6^ Department of Anus & Intestine Surgery, Key Laboratory of Diagnosis and Treatment of Digestive System Tumors of Zhejiang Province, Hua Mei Hospital, University of Chinese Academy of Sciences, Ningbo, China; ^7^ Department of Clinical Laboratory, The First Affiliated Hospital of Sun Yat-Sen University, Guangzhou, China; ^8^ Department of Global Health, Ningbo Institute of Life and Health Industry, University of Chinese Academy of Sciences, Ningbo, China

**Keywords:** non-alcoholic fatty liver disease, aortic calcification, propensity score-matching, Cox proportional-hazards regression, cohort study

## Abstract

**Objectives:**

Non-alcoholic fatty liver disease (NAFLD) greatly affects cardiovascular disease, but evidence on the associations between NAFLD and markers of aortic calcification is limited. We aim to evaluate the association between NAFLD and aortic calcification in a cohort of Chinese adults using propensity score-matching (PSM) analysis.

**Methods:**

This prospective cohort study involved adults who underwent health-screening examinations from 2009 to 2016. NAFLD was diagnosed by abdominal ultrasonography at baseline, and aortic calcification was identified using a VCT LightSpeed 64 scanner. Analyses included Cox proportional-hazards regression analysis and PSM with predefined covariates (age, gender, marital and smoking status, and use of lipid-lowering drugs) to achieve a 1:1 balanced cohort.

**Results:**

Of the 6,047 eligible participants, 2,729 (45.13%) were diagnosed with NAFLD at baseline, with a median age of 49.0 years [interquartile range, 44.0–55.0]. We selected 2,339 pairs of participants with and without NAFLD at baseline for the PSM subpopulation. Compared with those without NAFLD, patients with NAFLD were at a higher risk of developing aortic calcification during follow-up; significant results were observed before and after matching, with the full-adjusted hazard ratios and corresponding 95% confidence intervals being 1.19 (1.02–1.38) and 1.18 (1.01–1.38), respectively (both *p* < 0.05). In subgroup analyses, no interaction was detected according to age, gender, smoking status, body mass index, total cholesterol, low-density lipoprotein cholesterol, use of lipid-lowering drugs, hypertension, or type 2 diabetes.

**Conclusions:**

NAFLD may be independently associated with aortic calcification. Further studies are warranted to elucidate the possible underlying mechanisms.

## Introduction

As the global epidemic of obesity has fueled metabolic conditions, the burden of non-alcoholic fatty liver disease (NAFLD) has become enormous (1). Globally, NAFLD prevalence steadily increased from 15% in 2005 to 25% in 2010, with the highest prevalence observed in the Middle East (32%) and the lowest in Africa (14%) (1–3). As one of the most prevalent causes of disease, NAFLD has aroused critical concern worldwide (4). NAFLD appears to increase the burden of subclinical atherosclerosis (5), which is closely related to the development of vascular calcification. Several mechanisms may be involved in the acceleration of vascular calcification in patients with NAFLD, including insulin resistance, dyslipidemia, inflammation, oxidative stress, and imbalance of adipokines and coagulation (6, 7). Given the upward trend in the prevalence of NAFLD, it is necessary to elucidate the relationship between NAFLD and vascular calcification.

In a cross-sectional study conducted in Korea (8), NAFLD was associated with the presence of coronary artery calcification (CAC) among young and middle-aged individuals, even after adjustment for cardiovascular and metabolic risk factors (multivariable-adjusted odd ratio [OR] = 1.10; 95% confidence interval [CI] = 1.05–1.16). In another retrospective cohort study of 4,731 adults in Korea (9), the annual rate of CAC progression was significantly higher in participants with NAFLD than those without NAFLD (22% vs. 17%; *p* < 0.001), and the multivariable ratio of progression rates comparing NAFLD to non-NAFLD was 1.04 (CI = 1.02–1.05; *p* < 0.001).

However, findings from previous observational studies were influenced by covariates. For instance, in a cross-sectional study in Korea, neither CAC nor calcified plaque were significantly associated with NAFLD (*p* = 0.375 and 0.214, respectively) after adjustment for multiple cardiovascular risk factors (i.e., age, gender, hypertension, hyperlipidemia, diabetes mellitus, current smoking, history of coronary artery disease, and level of high-sensitivity C-reactive protein [hs-CRP]) (10). An American cross-sectional study investigated the associations between NAFLD and calcification in eight different vascular beds; however, significant associations were limited to calcification of the thoracic aorta (multivariable-adjusted OR = 1.38; CI = 1.09–1.78) and celiac trunk (multivariable-adjusted OR = 2.05; CI = 1.16–3.65) after adjusting for traditional risk factors of chronic venous disease (11). Similarly, another study in the United States found liver attenuation (LA), which decreases as liver fat increases, and CAC to be significantly correlated in models adjusted for age and gender and models adjusted for multiple variables (both *p*-values < 0.05). In contrast, the association between LA and abdominal aortic calcification (AAC) only persisted in models adjusted for age and gender (12). Furthermore, in a study in Netherlands, Wolff et al. reported that higher LA was associated with a lower volume of epicardial fat and decreased risk of CAC, independent of cardiovascular risk factors. They also observed, however, strong associations between covariates (waist circumference, diastolic blood pressure, and diabetes) and lower LA, with multivariable-adjusted beta values of −2.54, −0.52, and −21.91, respectively (13). The inconsistency of these results highlights the importance of investigating these associations while controlling the distribution of confounders.

Hence, we aim to evaluate the relationship between having NAFLD at baseline and aortic calcification in a large prospective Asian cohort using propensity score matching (PSM) analysis, which was invented to help ensure an even distribution of confounders between groups and increase intergroup comparability (14).

## Materials and Methods

### Study Design and Participants

This cohort included 52,402 participants who underwent an annual or biennial physical examination at the clinic of Xiao Tang Shan Hospital (Beijing, China) from January 1, 2009 to December 31, 2016. Further details of this cohort were previously described (15). We excluded participants who were under 20 years old, died during the follow-up period, or completed less than two follow-ups. In this study, the first measurement of data was considered the baseline. The principal exposure under investigation was diagnosed NAFLD versus non-NAFLD, and the primary outcome was the occurrence of aortic calcification during the follow-up period. Hence, participants who were missing information related to the diagnosis of NAFLD and/or aortic calcification were also excluded.

Participant enrollment for this cohort conformed to the ethical guidelines of the 1975 Declaration of Helsinki and was approved by the Institutional Review Board of Xiao Tang Shan Hospital. This study was based on routine health-screening data without identification information; computed tomography (CT) scans for aortic calcification have also been regarded as part of a routine examination. Thus, the requirement of informed consent was waived in our study.

### Data Measurements

During a comprehensive health checkup, demographic factors, information about smoking and alcohol consumption, past medical and pharmaceutical records, and family history of disease were collected by well-trained research assistants through face-to-face interviews in the beginning of our study. Physical measurements (e.g., anthropometric and laboratory data) were recorded using standard methods by nurses or physicians at baseline and updated every year at the annual follow-up.

After overnight fasting, laboratory evaluations of the lipid profile, liver function, and serum glucose were conducted using an enzymatic colorimetric assay (Type 7600, Hitachi, Tokyo), the glucose dehydrogenase method (Merck, Darmstadt, Germany), and an automated analyzer, respectively. The presence of type 2 diabetes mellitus (T2DM) was indicated by (1) a self-report of diabetes diagnosed by a physician, (2) antidiabetic drugs having been used within the last 2 weeks, (3) a fasting blood glucose (FPG) ≥7.0 mmol/L, (4) a 2-h postprandial plasma glucose ≥11.1 mmol/L, or (5) glycosylated hemoglobin ≥48.0 mmol/mol (6.5%). Hypertension was defined as a routine blood pressure measurement that reached 140/90 mmHg or higher or having self-reported the use of anti-hypertensive drugs within the previous 2 weeks at study entry.

### Ascertainment of NAFLD and Aortic Calcification

NAFLD was diagnosed based on ultrasonographic features and the absence of potential causes of fat accumulation in the liver, particularly excessive daily alcohol consumption (≥30 g/day for men and ≥20 g/day for women) (16). To detect potential pathological changes linked to fatty liver, an abdominal ultrasound examination was performed by experienced radiologists using the HD7 ultrasound system (Philips, Shenyang, China). The presence of fatty liver disease should be confirmed if at least two of the following three abnormal manifestations are present: (1) parenchymal brightness, (2) deep beam attenuation and bright vessel walls, and (3) increased liver–kidney contrast (17).

Aortic calcification was detected using a VCT LightSpeed 64 scanner (GE Healthcare, Tokyo, Japan) at the medical center of Xiao Tang Shan Hospital following a standard scanning protocol: 2.5 mm thickness, 400 ms rotation time, 120 kV tube voltage, and 124 mAs (310 mA × 0.4 s) tube current under ECG-gated dose modulation (8). The scans were analyzed on Advantage Workstations (GE Healthcare) by experienced radiologists. The image of each bed was obtained by scanning segments with the following slice thicknesses: 3 mm for the vascular walls of the coronary artery, 5 mm in the thoracic aorta, and 6 mm through the neck, abdominal, and pelvic beds. Calcification was identified as a plaque whose area was >50% calcified tissue with a density of more than 130 Hounsfield units (10).

### Statistical Analysis

Data normality was assessed using a Kolmogorov–Smirnov test with Dallal–Wilkinson–Lillie corrected *p*-values. Non-parametric data are described as medians and interquartile ranges (IQR); categorical data are expressed as numbers with the corresponding proportions. Differences in baseline characteristics between the non-NAFLD and NAFLD groups were evaluated using the Wilcoxon rank-sum test for nonparametric variables and a chi-square test for categorical variables.

PSM analysis was conducted to minimize residual confounders and reduce bias between non-NAFLD and NAFLD groups (18). Covariates were chosen for PSM based on two criteria: (1) being unrelated to the exposure/treatments and (2) being known to affect the risk of the outcome (19). Based on current evidence (8–11, 20, 21), the covariates included age, gender, marriage, smoking status, hypercholesterolemia, hypertension, and T2DM. The inclusion of too many covariates could reduce the number of good matches and decrease the precision (22). **Supplementary Material** are available on Figshare (doi: 10.6084/m9.figshare.16570410.v1). Based on the results of Spearman’s rank correlation analysis and the results of a balance test that compared the distribution of confounders in the matched samples, only five baseline covariates (age, gender, marital status, smoking status, and use of lipid-lowering drugs) were selected for PSM (**Supplementary Table S1**). The propensity score was calculated for each participant using a multivariate logistic regression model, and participants were assigned evenly to the NAFLD and non-NAFLD groups using the greedy nearest-neighbor method with a caliper width equal to 0.02 (23). A standardized difference between −0.10 and 0.10 indicated an adequate balance (24).

Cox proportional-hazards regression analysis was conducted to estimate the hazard ratio (HR) with a 95% CI for aortic calcification associated with baseline NAFLD for the unmatched and matched populations. Before PSM, a series of models was utilized to assess the explanatory variables for the samples: Model 1 was crude analysis without adjustment; Model 2 was adjusted for age, gender, marital status, smoking status, systolic blood pressure (SBP), diastolic blood pressure (DBP), FPG, alanine transaminase (ALT), and aspartate aminotransferase (AST); Model 3 was further adjusted for low-density lipoprotein cholesterol (LDL-C) and the use of anti-hypertensive and lipid-lowering drugs; finally, Model 4 was adjusted for waist and hip circumference. Incomplete matching could lead to biased results (25); imbalanced covariates were double adjusted to reduce potential confounding effects (24, 26). After PSM, the following models were applied: Model 5 was adjusted for imbalanced covariates with *p-*values <0.05 after matching (i.e., age and smoking status); Model 6 was further adjusted for FPG, SBP, DBP, ALT, and AST; Model 7 was adjusted for the covariates in model 6 plus LDL-C, using the statuses of anti-hypertensive drugs and lipid-lowing drugs (Yes vs. No); and Model 8 was further adjusted for waist and hip circumference to evaluate the potential mediation of fat mass in the association between NAFLD and aortic calcification.

Finally, subgroup analyses stratified by age, gender, smoking status, body mass index (BMI), total cholesterol (TC), LDL-C, the use of lipid-lowering drugs, hypertension, and T2DM were further conducted for both unmatched and matched samples to assess whether the association changed according to these settings. Missing data were treated as unable to affect other variables. All analyses were performed using the software R 4.0.5 (R Development Core Team, Vienna, Austria); a two-sided *p* < 0.05 was considered statistically significant.

## Results

### Study Population

**Figure 1:**
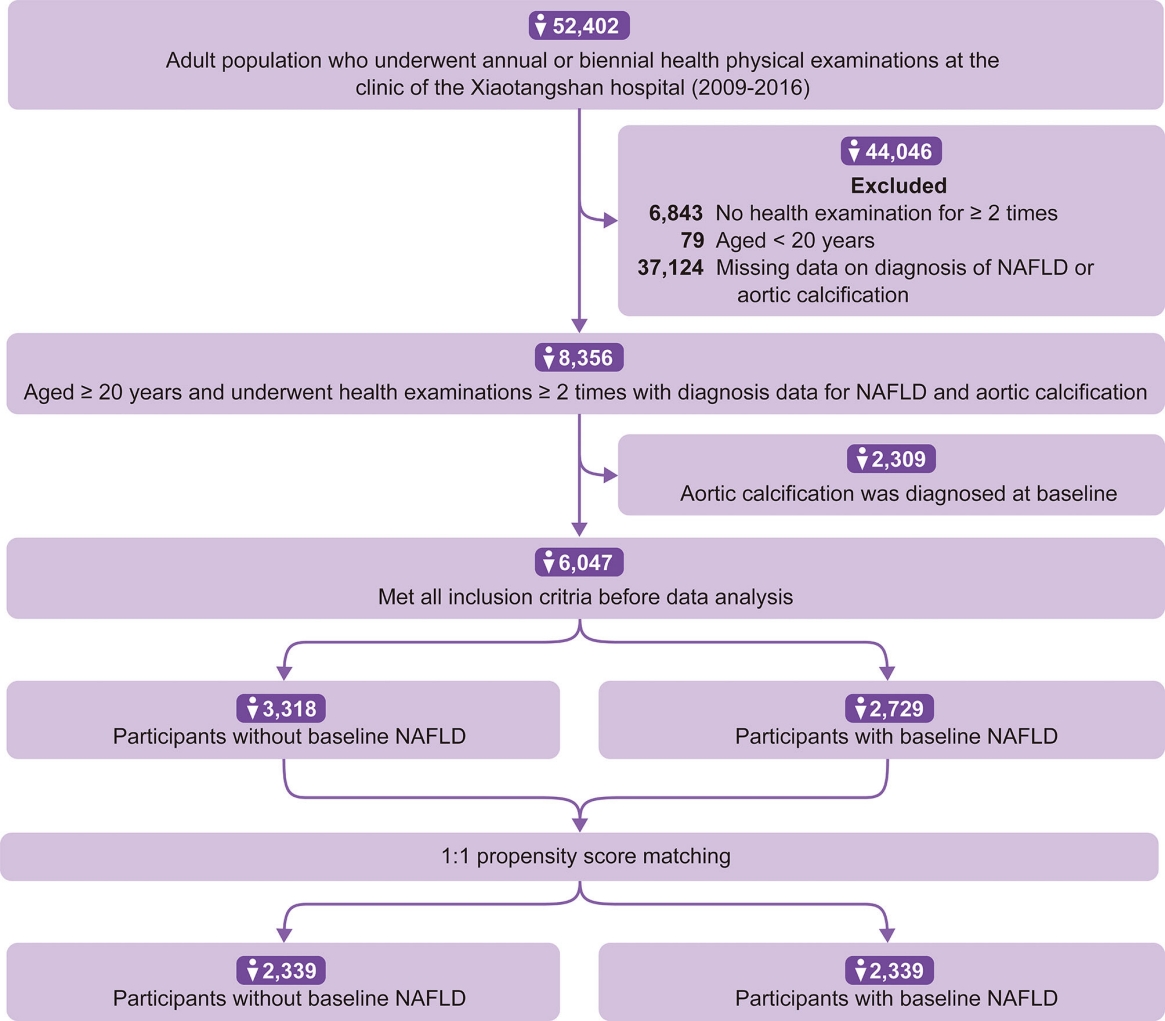
Flow chart of study participants selection.

We identified 52,402 participants who underwent baseline physical examinations; 46,355 participants were excluded for not undergoing a follow-up examination (n = 6,843), being younger than 20 years old (n = 97), or having no diagnosis of NAFLD or aortic calcification (n = 37,124). We further excluded 2,388 participants who were diagnosed with aortic calcification at baseline. As a result, 6,047 participants were included for analysis (**Figure 1**).

### Baseline Characteristics

Of the 6,047 eligible participants, 4,148 (68.6%) were male. The median age at baseline was 48.0 years (IQR = 42.0–55.0). At the follow-up examination, we identified 836 participants (13.8%) with newly diagnosed aortic calcification. The 2,729 (45.1%) patients with NAFLD at baseline were older than the 3,318 (54.9%) non-NAFLD patients (49.0 vs. 47.0 years; *p* < 0.001), and a greater proportion were male (80.6% vs. 58.7%; *p* < 0.001) (**Table 1**). Significant between-group differences were also observed in marital status, smoking status, use of lipid-lowering drugs, TC, LDL-C, high-density lipoprotein cholesterol, triglycerides, ALT, AST, BMI, waist circumference, hip circumference, SBP, DBP, hypertension, use of anti-hypertensive drugs, FPG, T2DM, uric acid, creatinine (Cr), blood urea nitrogen, the number of follow-up examinations taken as part of this study, and the number of cases of aortic calcification (all *p* values < 0.05, **Table 1**). After matching for age, gender, marital status, and smoking status, 2,339 non-NAFLD and NAFLD pairs were identified (**Table 1**; **Supplementary Figures S1, S2**). The distribution of gender, marital status, and use of lipid-lowering drugs between the groups was adequately balanced (standardized differences = 0.00, all *p*-values between groups > 0.05). The distribution of age between groups nearly achieved balance (standardized difference = 0.09; *p* between groups < 0.001); an imbalance was still observed for smoking status (standardized differences ranged from −0.16 to 0.06; *p* between groups < 0.001) (**Table 1**). As shown in **Table 1**, aside from the level of Cr (*p* = 0.349), the differences between the groups for other unmatched covariates at baseline remained significant (all *p*-values < 0.05).

**Table 1:** Comparison of baseline variables between participants with and without non-alcoholic fatty liver disease in the unmatched and matched population^a^.

Characteristic	Unmatched population (n = 6,047)			Matched population (n = 4,678)			Standardized differences^b^		
	Non-NAFLD (n=3,318)	NAFLD (n=2,729)	*p*-value^c^	Non-NAFLD (n=2,339)	NAFLD (n=2,339)	*p*-value^c^	Unmatched	Matched	Balance improvement (%)
Age, y	47.0 (41.0–55.0)	49.0 (44.0–55.0)	<0.001	49.0 (43.0–55.0)	50.0 (45.0–56.0)	<0.001	0.13	0.09	27.9
Gender, No. (%)			<0.001			1.000			
Male	1,948 (58.7)	2,200 (80.6)		1,818 (77.7)	1,818 (77.7)		0.55	−0.00	100.0
Female, No. (%)	1,370 (41.3)	529 (19.4)		521 (22.3)	521 (22.3)		−0.55	0.00	100.0
Marital status, No. (%)			<0.001			0.977			
Yes	3,072 (97.3)	2,606 (99.1)		2,228 (95.3)	2,229 (95.3)		0.14	0.00	98.5
No	246 (2.8)	123 (0.9)		20 (0.9)	21 (0.9)		−0.19	−0.00	97.5
Smoking status, No. (%)			<0.001			<0.001			
Never	2,148 (64.7)	1,444 (52.9)		1,291 (55.2)	1,363 (58.3)		−0.24	0.06	73.9
Past	585 (17.6)	645 (26.6)		520 (22.2)	602 (25.7)		0.14	0.08	41.6
Current	586 (17.7)	640 (23.5)		528 (22.6)	374 (16.0)		0.14	−0.16	−13.7
Use of lipid-lowing drugs, No. (%)			0.004			1.000			
Yes	33 (1.0)	52 (1.9)		25 (1.1)	26 (1.1)		0.07	0.00	95.3
No	3,285 (99.0)	2,677 (98.1)		2,314 (98.9)	2,313 (98.9)		−0.07	−0.00	95.3
Total cholesterol, mmol/L	4.83 (4.27–5.45)	5.07 (4.48–5.68)	<0.001	4.92 (4.34–5.54)	5.07 (4.48–5.69)	<0.001	0.24	–	
LDL-C, mmol/L	2.98 (2.49–3.46)	3.19 (2.67–3.70)	<0.001	3.01 (2.56–3.51)	3.18 (2.67–3.69)	<0.001	0.24	–	
HDL-C, mmol/L	1.38 (1.17–1.62)	1.17 (1.04–1.36)	<0.001	1.35 (1.15–1.58)	1.18 (1.04–1.38)	<0.001	−0.81	–	
Triglycerides, mmol/L	1.13 (0.83–1.58)	1.89 (1.38–2.66)	<0.001	1.23 (0.91–1.70)	1.87 (1.36–2.60)	<0.001	0.57	–	
ALT, U/L	17.1 (13.3–23.5)	26.0 (19.7–36.2)	<0.001	18.6 (14.1–25.0)	25.8 (19.0–36.0)	<0.001	0.50	–	
AST, U/L	19.0 (16.8–23.0)	21.6 (18.0–26.2)	<0.001	19.6 (16.8–23.0)	21.0 (18.0–26.0)	<0.001	0.26	–	
BMI, kg/m^2^	24.1 (22.2–25.8)	27.3 (25.6–29.1)	<0.001	24.4 (22.8–26.1)	27.2 (25.5–29.1)	<0.001	1.00	–	
Waist circumference, cm	80.0 (72.0–87.0)	91.0 (84.0–97.0)	<0.001	83.0 (75.0–89.0)	91.0 (83.0–96.0)	<0.001	0.28	–	
Hip circumference, cm	94.0 (90.0–98.0)	99.0 (94.0–103.0)	<0.001	95.0 (90.0–98.0)	99.0 (94.0–102.0)	<0.001	0.13	–	
SBP, mmHg	116.0 (108.0–127.0)	124.0 (116.0–135.0)	<0.001	118.0 (110.0–128.0)	123.0 (115.0–134.0)	<0.001	0.42	–	
DBP, mmHg	72.0 (66.0–80.0)	79.0 (71.0–85.0)	<0.001	75.0 (70.0–80.0)	78.0 (70.0–85.0)	<0.001	0.44	–	
Hypertension, No. (%)			<0.001			<0.001			
Yes	710 (21.4)	1,139 (41.7)		578 (24.7)	974 (41.6)		0.41	–	
No	2,608 (78.6)	1,590 (58.3)		1,761 (75.3)	1,365 (58.4)		–0.41	–	
Use of anti-hypertensive drugs, No. (%)						0.011			
Yes	119 (3.6)	168 (6.2)		92 (3.9)	130 (5.6)		0.11	–	
No	3.199 (96.4)	2,561 (93.8)		2,247 (96.1)	2,209 (94.4)		−0.11	–	
FPG, mmol/L	5.15 (4.86–5.52)	5.52 (5.17–6.10)	<0.001	5.19 (4.87–5.57)	5.52 (5.15–6.09)	<0.001	0.50	–	
T2DM, No. (%)			<0.001			<0.001			
Yes	224 (6.8)	459 (16.8)		178 (7.6)	396 (16.9)		0.27	–	
No	3,094 (93.2)	2,270 (83.2)		2,161 (92.4)	1,943 (83.1)		–0.27	–	
UA, umol/L	319.9 (260.5–378.9)	381.3 (329.0–436.0)	<0.001	343.0 (292.0–394.2)	378.8 (327.0–432.1)	<0.001	0.73		
Cr, umol/L	79.5 (67.8–90.7)	84.0 (73.6–93.6)	<0.001	84.5 (72.4–93.9)	84.0 (73.2–93.9)	0.349	0.34		
BUN, mmol/L	4.80 (4.00–5.70)	5.09 (4.30–5.90)	<0.001	5.00 (4.21–5.86)	5.09 (4.30–5.90)	0.027	0.27		
Total person-year of follow-up (year)	2.70 (1.03–5.18)	2.12 (1.03–4.48)	0.004	2.93 (1.05–5.88)	2.38 (1.04–4.94)	<0.001	−0.10	–	
Aortic calcification cases			<0.001			0.003			
Yes	403 (12.16)	433 (15.87)		330 (14.11)	405 (17.32)		0.10	–	
No	2,915 (87.85)	2,296 (84.13)		2,009 (85.89)	1,935 (82.68)		−0.10	–	

a All continuous variables failed to pass the normality test, therefore, were described as median with IQR.

b Difference in mean levels between non-NAFLD and NAFLD groups, and standardized difference between −0.10 and 0.10 indicated an adequate balance.

c p-value for between group difference was calculated using Wilcoxon test for continuous variables and Chi-square test for categorical variables.

ALT, alanine aminotransferase; AST, aspartate aminotransferase; BUN, blood urea nitrogen; BMI, body mass index; Cr, creatinine; DBP, diastolic blood pressure; FPG, fasting plasma glucose; HDL-C, high density lipoprotein cholesterol; IQR, inter-quantiles range; LDL-C, low density lipoprotein cholesterol; NAFLD, nonalcoholic fatty liver disease; PSM, propensity scoring matching; SBP, systolic blood pressure; T2DM, type 2 diabetes mellitus; UA, uric acid.

### Association Between Baseline NAFLD and Aortic Calcification Risk

For the unmatched population, crude analysis indicated that participants with NAFLD were at higher risk for aortic calcification than the non-NAFLD group (HR = 1.38; 95% CI = 1.21–1.58; *p* < 0.001). After additional adjustment for other covariates (Models 2–4), increased risk of aortic calcification was still observed in NAFLD participants compared with non-NAFLD participants (Model 2: HR = 1.24; 95% CI = 1. 08–1.43; *p* = 0.003, Model 3: HR = 1.21; 95% CI = 1.04–1.39; *p* = 0.001, Model 4: HR = 1.19; 95% CI = 1.02–1.38; *p* = 0.023) (**Table 2**). After PSM, we adjusted imbalanced variables (age and smoking status) in Model 5 and performed multiple adjusted models to explore the influence of unmatched variables on the association between baseline NAFLD and aortic calcification risk. The results from the matched population were consistent with findings from the unmatched population: baseline NAFLD was independently associated with an increased risk of aortic calcification (Model 5: HR = 1.32; 95% CI = 1.14–1.52; *p* < 0.001, Model 6: HR = 1.22; 95% CI = 1.05–1.43; *p* = 0.009, Model 7: HR = 1.20; 95% CI = 1.03–1.40; *p* = 0.020, Model 8: HR = 1.18; 95% CI, 1.01–1.38; *p* = 0.036) (**Table 2**).

**Table 2:** Association between non-alcoholic fatty liver status at baseline and risk of aortic calcification^a^.

	Before PSM			After PSM	
	HR (95%CI)	*p*-value		HR (95%CI)	*p*-value
**Model 1**			**Model 5**		
Non-NAFLD	1.00 (Ref)		Non-NAFLD	1.00 (Ref)	
NAFLD	1.38 (1.21, 1.58)	**<0.001**	NAFLD	1.32 (1.14, 1.52)	<0.001
**Model 2**			**Model 6**		
Non-NAFLD	1.00 (Ref)		Non-NAFLD	1.00 (Ref)	
NAFLD	1.24 (1.08, 1.43)	**0.003**	NAFLD	1.22 (1.05, 1.43)	0.009
**Model 3**			**Model 7**		
Non-NAFLD	1.00 (Ref)		Non-NAFLD	1.00 (Ref)	
NAFLD	1.21 (1.04, 1.39)	**0.011**	NAFLD	1.20 (1.03, 1.40)	0.020
**Model 4**			**Model 8**		
Non-NAFLD	1.00 (Ref)		Non-NAFLD	1.00 (Ref)	
NAFLD	1.19 (1.02, 1.38)	**0.023**	NAFLD	1.18 (1.01, 1.38)	0.036

a HR with 95% CIs was calculated using Cox proportional hazards regression analysis.

Before PSM, model 1 was crude analysis; model 2 was initially adjusted for age and gender, marital status (Yes vs. No), smoking status (never, past, and current), SBP (mmHg), DBP (mmHg), FPG (mmol/L), ALT (U/L), and AST (U/L); model 3 was further adjusted for LDL-C (mmol/L), anti-hypertensive drugs (Yes vs. No), and lipid-lowing drugs (Yes vs. No); model 4 was further adjusted for waist circumference (cm) and hip circumference (cm). After PSM, model 5 was adjusted for unbalanced variables (age and smoking status); model 6 was further adjusted for FPG (mmol/L), SBP (mmHg), DBP (mmHg), ALT (U/L), and AST (U/L); model 7 was adjusted covariates in model 6 plus LDL-C (mmol/L), anti-hypertensive drugs (Yes vs. No), and lipid-lowing drugs (Yes vs. No); and model 8 was further adjusted for waist circumference (cm), hip circumference (cm).

ALT, alanine aminotransferase; AST, aspartate aminotransferase; CI, confidence interval; DBP, diastolic blood pressure; FPG, fasting plasma glucose; HR, hazard ratio; NAFLD, non-alcoholic fatty liver disease; PSM, propensity scoring matching; SBP, systolic blood pressure; LDL-C, low density lipoprotein cholesterol.

### Subgroup and Sensitivity Analyses

To evaluate the potential effects of other clinical characteristics on the risk of aortic calcification, subgroup analyses were conducted on both the unmatched and matched population (**Supplementary Table S2**; **Table 3**). We stratified by age (20.0–44.9, 45.0–64.9, and ≥65.0 years), gender (male and female), smoking status (never, past, and current), BMI (< 24.0, 24.0–27.9 and ≥ 28.0 kg/m^2^), TC (< 4.99 and ≥ 4.99 mmol/L; < 5.00 and ≥ 5.00 mmol/L), LDL-C (< 3.09 and ≥ 3.09 mmol/L; < 3.13 and ≥ 3.13 mmol/L), use of lipid-lowering drugs (yes and no), and comorbid hypertension or T2DM (yes and no), and found no statistically significant interaction effects before and after PSM (*p* for interactions ranged from 0.172 to 0.967). Given that patients with viral hepatitis were not excluded at the beginning of our study, we conducted sensitivity analysis by excluding 69 (2.99%) and 6 (0.23%) participants who were infected with Hepatitis B virus and Hepatitis C virus, but associations between baseline NAFLD and AC remained significant in full-adjusted models 4 and 8 (data no shown).

**Table 3:** Subgroup analyses of variables in relation to non-alcoholic fatty liver disease with the incidence of aortic calcification after propensity score matching^a^.

Characteristics	Number	Total person-year of follow-up (year)	Aortic calcification cases	HR (95% CI)		*p*-value	*p* for interaction
				Non-NAFLD	NAFLD		
Age (year)							0.172
20.0–44.9	1,274	3,705.2	33	1.00	1.08 (0.49, 2.40)	0.849	
45.0–64.9	3,076	10,725.7	554	1.00	1.22 (1.02, 1.47)	0.031	
≥65.0	328	926.5	148	1.00	1.05 (0.73, 1.51)	0.784	
Gender							0.741
Male	3,636	11,370.0	570	1.00	1.15 (0.97, 1.37)	0.110	
Female	1,042	3,987.5	165	1.00	1.33 (0.93, 1.90)	0.117	
Smoking status							0.825
Never	2,654	8,986.6	401	1.00	1.26 (1.02, 1.55)	0.032	
Past	1,122	3,479.0	179	1.00	1.01 (0.73, 1.40)	0.956	
Current	902	2,891.9	155	1.00	1.10 (0.77, 1.56)	0.617	
BMI (kg/m^2^)^b^							0.938
<24.0	1,197	4,223.9	158	1.00	1.25 (0.80, 1.95)	0.324	
24.0–27.9	2,421	7,879.1	366	1.00	1.07 (0.86, 1.32)	0.561	
≥28.0	1,060	3,254.5	211	1.00	1.00 (0.70, 1.45)	0.981	
Total cholesterol (mmol/L)							0.668
<5.04^c^	2,435	7,636.2	299	1.00	1.25 (0.99, 1.59)	0.065	
≥5.04	2,243	7,721.3	436	1.00	1.17 (0.95, 1.45)	0.077	
LDL-C (mmol/L)							0.711
<3.13^d^	2,387	8,075.7	330	1.00	1.22 (0.97, 1.53)	0.088	
≥3.13	2,291	7,281.8	405	1.00	1.16 (0.93, 1.44)	0.186	
Use of lipid-lowering drugs							0.724
Yes	51	81.1	4	1.00	0.03 (0.00, 7.70)	0.218	
No	4,627	15,276.4	731	1.00	1.21 (1.04, 1.42)	0.014	
Hypertension							0.333
Yes	1,552	4,822.7	339	1.00	1.15 (0.93, 1.42)	0.186	
No	3,126	10,534.8	396	1.00	1.13 (0.89, 1.43)	0.315	
T2DM							0.858
Yes	574	1,758.2	111	1.00	1.18 (0.76, 1.82)	0.468	
No	4,104	13,599.3	624	1.00	1.14 (0.96, 1.35)	0.139	

^a^All subgroup analyses were conducted using maximally adjusted model (Model 8).

^b^BMI was grouped according to the BMI standard of Chinese population.

^c^5.04 mmol/L was the mean value of total cholesterol among all matched participants.

^d^3.13 mmol/L was the mean value of LDL-C among all matched participants.

BMI, body mass index; CI, confidence interval; HR, hazard ratio; NAFLD, nonalcoholic fatty liver disease; LDL-C, low density lipoprotein cholesterol; T2DM, type 2 diabetes mellitus.

## Discussion

In this large cohort study of 6,047 middle-aged Chinese adults, the NAFLD group had a significantly higher risk of aortic calcification than the non-NAFLD group, independent of established covariates (gender, age, smoking status, BMI, TC, LDL-C, use of lipid-lowering drugs, hypertension, and T2DM). A significant association was still observed after PSM and multivariable adjustment, which substantiated the credibility of our findings by mimicking the randomization of a prospective study and reducing bias caused by confounding variables (18).

Despite limited evidence directly linking NAFLD to aortic calcification among the population, an increased risk of atherosclerotic calcification associated with NAFLD has been reported previously. For instance, a recent systematic review provided supporting evidence that pathophysiological mechanisms of NAFLD, such as a proinflammatory state and an increase in oxidative stress, were closely related with increased risk of cardiovascular diseases (CVDs) (27). Specially, in a retrospective cohort study of 4,731 Korean adults with no history of CVD at the Samsung Medical Center’s Health Promotion Center (2004–2013), CAC scores, which are a validated measure of risk for coronary heart disease, were found to be significantly and positively associated with NAFLD, independent of cardiovascular and metabolic risk factors, with a multivariable-adjusted OR of 1.03 (95% CI = 1.02–1.05; *p* < 0.001) (9). Similar results were also seen in a later study, in which data were collected from 105,328 Korean adults at Kangbuk Samsung Hospital’s Healthcare Center (2011–2017), and a positive association between NAFLD and the presence of CAC was found (multivariable-adjusted OR = 1.10; 95% CI = 1.05–1.16). The results remained statistically significant in *post-hoc* analysis and after further adjustment for homeostasis assessment (insulin resistance and hs-CRP) (8). A study of 2,424 black and white young adults (the Coronary Artery Risk Development in Young Adults study) in the United States observed increased incidence of CAC (37.9% vs. 26.0%; *p* < 0.001) and AAC (65.1% vs. 49.9%; *p* < 0.001) among those with NAFLD, and significantly increased risks of CAC (OR = 1.33; 95% CI = 1.00–1.82) and AAC (OR = 1.74; 95% CI = 1.29–2.35) after adjustment for demographics and healthy behavior (28).

Atherosclerotic calcification has been regarded as the most common form of calcific vasculopathy, which includes arterial wall calcification in the aorta and coronary and peripheral arteries (29, 30). Although calcification is common in all atherosclerotic lesions, independent of location (coronary, aorta, and peripheral arteries) (31), small vessels such as the radial and digital vessels are more likely to be calcified directly, whereas calcifications in large arteries such as the coronary and carotid arteries are more prone to develop from atherosclerosis, i.e., the accumulation of fatty and/or fibrous material in the intima of many middle-sized and large arteries (31–33). Moreover, the association of NAFLD with calcification might depend on the vascular bed, as risk factors for calcified atherosclerosis differ between arterial beds (34). In a community-based cohort with CT scans of eight different vascular beds (carotid artery, coronary artery, thoracic aorta, abdominal aorta, iliac artery, renal artery, celiac trunk, and superior mesenteric artery), NAFLD was found to be significantly associated with calcifications in the coronary artery, carotid artery, thoracic aorta, celiac trunk, and superior mesenteric artery in models adjusted for age and gender (all *p-*values < 0.05) (11). However, most of these associations between NAFLD and calcification did not remain significant after adjustment for obesity, smoking status, hypertension, dyslipidemia, diabetes, and family history of heart disease. The exceptions were the thoracic aorta (multivariable-adjusted OR = 1.38; 95% CI = 1.09–1.78) and the celiac trunk (multivariable-adjusted OR = 2.05; 95% CI = 1.16–3.65) (11), indicating that traditional risk factors for chronic venous disease might confound the association between NAFLD and vascular calcification.

Moreover, in retrospective observational studies, confounders tend to differ between groups; differences in outcomes might reflect differences in baseline data rather than treatment effects (22). For example, in a community-based longitudinal study in the United States, a significant association between NAFLD and increased risk of subclinical atherosclerosis (CAC and AAC) was attenuated after adjusting for visceral adipose tissue (VAT), a marker of obesity; the multiple-adjusted ORs and 95% CIs became 1.05 (0.74–1.48) for CAC and 1.20 (0.86–1.67) for AAC (28). Furthermore, in a study involving African Americans, fatty liver was associated with CAC independent of traditional risk factors, whereas the association between VAT and CAC was diminished after multivariable adjustments (multiple-adjusted OR = 1.10, 95% CI = 0.9–1.2, *p* = 0.09); AAC was associated with LA and VAT only in the model adjusted for age and gender (both *p* < 0.05) (12). Although Wolff et al. found an association between liver fat and CAC, they reported positive associations between waist circumference, DBP, diabetes, and larger volumes of fatty liver (13). In addition, a study involving 1,004 adults from the Multi-ethnic Study of Atherosclerosis (MESA) reported greater prevalence of AAC among black people with NAFLD than their white counterparts, regardless of gender (prevalence ratio = 1.41; 95% CI = 1.15–1.74; *p-*interaction = 0.02). Significant concurrent interactions between race and gender (*p-*interaction for Chinese vs. white people = 0.017; *p-*interaction for black people vs. white people = 0.042) were also found for the relationship between NAFLD and increased risk of AAC, indicating disparities in the development of atherosclerosis-related diseases (35).

In the current study, PSM was used to minimize confounding by balancing the confounders at baseline between groups (22). Nonetheless, dissimilarity in covariates between matched pairs is still a concern because PSM does not address the lack of comparability but instead creates a balanced distribution of all confounders (14). Cox proportional-hazards regression analysis and subgroup analyses were conducted for potential confounders after using the PSM model. We found an independent association between NAFLD and aortic calcification without interaction of potential confounders for both the matched and unmatched populations. These findings are similar to the results of a cross-sectional study from MESA, which suggested that NAFLD might be associated with CAC, independent of traditional risk factors (e.g., obesity or metabolic syndrome) (36).

The mechanisms by which NAFLD increases the risk of aortic calcification are not fully known. In consistent with our findings, Mario Masarone, et al. (37) also reported that most of the NAFLD patients tended to have T2DM due to the pathophysiological correlation with insulin resistance. T2DM has also been known as closely associated with risk of CAC and AAC due to its specific advanced glycation end products and metabolic oxidation products (38). The liver is an essential metabolic organ that contributes to systemic inflammation by secreting inflammatory markers, chemokines, and cytokines (e.g., tumor necrosis factor-α, interleukin-6, chemokine (C–C motif) ligand 3, soluble intercellular adhesion molecule-1, and hs-CRP), and can adversely affect the cardiovascular system through endothelial dysfunction, enhanced plaque formation, and altered vascular tone and coagulation (39). The abnormal release of cytokines associated with NAFLD could also disturb the balance between free radical activity and antioxidant activity and increase the level of oxidative stress (40). Yet, the liver also regulates lipid metabolism (41), which is one of the therapeutic targets for preventing calcification of the aortic intima (42). Recent studies have suggested that oxidized, medium-chain lipid peroxide-derived dicarboxylic acids are present in the lipid-rich domain of lesions, where they may bind to calcium and induce calcification of the aortic smooth-muscle cells upon intracellular micelle delivery before forming insoluble complexes (43). Notably, the altered level of oxidized low-density lipoprotein due to NAFLD could promote the migration of smooth muscle cells, thereby affecting atherosclerotic plaque vulnerability (43). Arterial calcification may be an attempt to protect the weakened atherosclerotic plaque prone to rupture (44); hence, calcification could potentially be regarded as a stabilizing force that can increase the biochemical stability of the plaque by imparting rigidity while at the same time decreasing the plaque’s mechanical stability (43). However, additional evidence is needed to clarify the potential mechanism underlying the relationship between NAFLD and aortic calcification.

Several limitations should be considered when interpreting our results. First, selection bias was inevitable in the current study due to the single-center design and lack of random enrollment of participants. Second, the NAFLD cases in our study were measured using the ultrasound rather than liver biopsy, the diagnostic gold standard of NAFLD that is not feasible given for those attending regular health screening examinations, suggesting the potential risk of measurement bias. Third, unmeasured or unknown residual confounders might remain even after PSM analysis. To mitigate potential bias, unmatched covariates were adjusted by hazard regression after PSM, and subgroup analyses were consequently performed to explore any unclear confounders. Fourth, participant data might be missing for relevant confounders, and the statistical power for detecting potential differences in some strata might be limited. Finally, as this cohort study involved Chinese adults, the applicability of our findings to other ethnic populations might be limited. Despite these limitations, as few studies have focused on the relationship between NAFLD and aortic calcification, the current study remains a meaningful contribution to this topic.

This cohort study indicates that there is an association between NAFLD at baseline and the risk of aortic calcification in the Chinese adult population. Effective screening and management of NAFLD is a potential approach for preventing atherosclerotic calcification in arteries. Further investigations are needed to support these findings.

## Data Availability Statement

The original contributions presented in the study are included in the article/**Supplementary Material**. Further inquiries can be directed to the corresponding authors.

## Ethics Statement

The studies involving human participants were reviewed and approved by the Institutional Review Board of Xiao Tang Shan Hospital. The patients/participants provided their written informed consent to participate in this study.

## Author Contributions

L-YH, P-NF, and X-YD were responsible for study concept and design. R-RZ, X-PG, and M-QL conceived the manuscript. Y-FC, S-XT, F-FZ, and Y-ML performed the quality control and revised it critically for important intellectual content. C-YW, SX, X-LP, and S-HD contributed to the analysis plan and interpretations. DZ, LW, and PZ contributed to the formal data analysis. All authors contributed to the article and approved the submitted version.

## Funding

This study was funded by the Key Laboratory of Diagnosis, Treatment and Research of Digestive System Tumor of Zhejiang Province (2019E10020), Youth Top-notch Personal Project from Fangshan District Excellent Talent Cultivation Funds (Grant no. 2016000000007B001), the Natural Public Welfare Fund of Zhejiang Province (LGC20H160002), the Medical and Health Science and Technology Foundation of Zhejiang Province (2019KY595, 2018KY690, 2018KY699, 2017KY593, and 2017KY594), the Natural Science Foundation of Ningbo (2018A610368, 2017A610145, 2017A610158, and 2016A610135), the Public Welfare Foundation of Ningbo (2021S108), Key Projects of Ningbo Public Welfare Fund (20211JCGY020386), and Ningbo Medical Discipline (2022-B11).

## Conflict of Interest

The authors declare that the research was conducted in the absence of any commercial or financial relationships that could be construed as a potential conflict of interest.

## Publisher’s Note

All claims expressed in this article are solely those of the authors and do not necessarily represent those of their affiliated organizations, or those of the publisher, the editors and the reviewers. Any product that may be evaluated in this article, or claim that may be made by its manufacturer, is not guaranteed or endorsed by the publisher.

## Supplementary Material

The Supplementary Material for this article can be found online at Figshare: https://doi.org/10.6084/m9.figshare.16570410.v1
