# Corrigendum: Non-alcoholic fatty liver disease is associated with aortic calcification: a cohort study with propensity score matching

**DOI:** 10.3389/fendo.2023.1248377

**Published:** 2023-07-10

**Authors:** Rong-Rong Zhu, Xu-Ping Gao, Min-Qi Liao, Yun-Feng Cui, Si-Xian Tan, Fang-Fang Zeng, Yan-Mei Lou, Chang-Yi Wang, Shan Xu, Xiao-Lin Peng, Shu-Hong Dai, Dan Zhao, Li Wang, Zhao Ping, Xiao-Yu Dai, Pin-Ning Feng, Li-Yuan Han

**Affiliations:** ^1^ Department of Pharmacy, Hua Mei Hospital, University of Chinese Academy of Sciences, Zhejiang, China; ^2^ Department of Public Health and Preventive Medicine, School of Medicine, Jinan University, Guangdong, China; ^3^ Department of Child & Adolescent Psychiatry, Peking University Sixth Hospital (Institute of Mental Health), National Clinical Research Center for Mental Disorders and NHC Key Laboratory of Mental Health (Peking University Sixth Hospital), Beijing, China; ^4^ Department of Health Management, Beijing Xiao Tang Shan Hospital, Beijing, China; ^5^ Department of Non-communicable Disease Prevention and Control, Shenzhen Nanshan Center for Chronic Disease Control, Shenzhen, China; ^6^ Department of Anus & Intestine Surgery, Key Laboratory of Diagnosis and Treatment of Digestive System Tumors of Zhejiang Province, Hua Mei Hospital, University of Chinese Academy of Sciences, Ningbo, China; ^7^ Department of Clinical Laboratory, The First Affiliated Hospital of Sun Yat-Sen University, Guangzhou, China; ^8^ Department of Global Health, Ningbo Institute of Life and Health Industry, University of Chinese Academy of Sciences, Ningbo, China

**Keywords:** non-alcoholic fatty liver disease, aortic calcification, propensity score-matching, Cox proportional-hazards regression, cohort study

In the published article, there was an error in the Funding statement. The funding from the Public Welfare Foundation of Ningbo (2021S108) was missing. The correct Funding statement appears below.

## Funding

This study was funded by the Key Laboratory of Diagnosis, Treatment and Research of Digestive System Tumor of Zhejiang Province (2019E10020), Youth Top-notch Personal Project from Fangshan District Excellent Talent Cultivation Funds (Grant no. 2016000000007B001), the Natural Public Welfare Fund of Zhejiang Province (LGC20H160002), the Medical and Health Science and Technology Foundation of Zhejiang Province (2019KY595, 2018KY690, 2018KY699, 2017KY593,2017KY594), the Natural Science Foundation of Ningbo (2018A610368, 2017A610145, 2017A610158, 2016A610135), the Public Welfare Foundation of Ningbo (2021S108), Key Projects of Ningbo Public Welfare Fund (20211JCGY020386), and Ningbo Medical Discipline (2022-B11).

The authors apologize for this error and state that this does not change the scientific conclusions of the article in any way. The original article has been updated.

